# The Illinois State Board of Health, Attacking Quackery

**Published:** 1878-12

**Authors:** 


### The Illinois State Board of Health Attacking Quackery.

In Chicago, in October, Judge Williams rendered a decision, in the case of Aikin vs. The State Board of Health. The complainant belongs to a class known as "advertising doctors," and held a certificate granted by the State Board, which permitted him to practice, and placed him on the list of the recognized physicians of the State. Recently he was warned that unless he desisted in his method of bringing himself before the public, his license would be revoked. The complainant then commenced the present suit, asking for an injunction, restraining the board from taking any action of the character, setting up that the board had no authority under the statutes, and that his course had not been unprofessional in any sense. An effort was also made to show that the law under which the board came into existence was unconstitutional.

The advertisements of Aikin professed his ability to cure all manner of diseases, and a number of reputable phyisicians were brought into court to testify that in a great many instances they were false, and liable to mislead ignorant people. As it was this class to protect which the State law was especially framed, it was held that the board had a right to discipline any member of the profession, and to decide what was unprofessional conduct in one of their calling. The court held that a board of this character must necessarily be vested with large discretionary power, and in the proper exercise of it should not be controlled by individual tribunals. It had, therefore, a right, in considering the sanitary conditions of the people, to decide what was "unprofessional," and what was not. In this sense a physician may be guilty of unprofessional conduct, and not criminal. Independent of the right of the board to exercise its authority without the intervention of the courts, it had been shown that the complainant had made statements in his advertisements which had been considered by a number of eminent practitioners as false as well as disreputable.

In discussing the constitutionality of the statute, the court spoke at considerable length, and said that the right to practice medicine was not descendable from its possessor to his heir, and may be lost by misconduct or immorality on the part of the practitioner. (12 Miss. 270; 5 Ohio, 22; 50 Ala., 486.) As the right to practice is a mere statutory privilege, there is no reason why it should not be denied to one man and extended to another in the discretion of the legislature. (See 12 District 17 Cal., 547; 22 Cal., 321.) The prayer for the injunction was therefore denied.—*Med. and Surg. Reporter.*

